# *Eupatorium fortunei* Turcz.: An Updated Review on the Botany, Phytochemistry, Pharmacology, and Toxicology

**DOI:** 10.3390/molecules31071137

**Published:** 2026-03-30

**Authors:** Jian-Qiang Ma, Yan-Ping Sun, Tian-Yuan Wu, Hui-Yue Yuan, Xin-Lan Li, Hua Huang, Li-Hong Wu, Zhi-Bin Wang, Hai-Xue Kuang

**Affiliations:** Key Laboratory of Basic and Application Research of Beiyao (Ministry of Education), Heilongjiang University of Chinese Medicine, 24 Heping Road, Xiangfang District, Harbin 150040, China; majianqianghzy@163.com (J.-Q.M.); sunyanping_1@163.com (Y.-P.S.); 15590862375@163.com (T.-Y.W.); 15308592866@163.com (H.-Y.Y.); lixinlan67@163.com (X.-L.L.); huahuang0813@163.com (H.H.); yolandahong1024@163.com (L.-H.W.)

**Keywords:** *Eupatorium fortunei* Turcz., botany, phytochemistry, pharmacology, toxicology

## Abstract

*Eupatorium fortunei* Turcz. (*E. fortunei*), a member of the Asteraceae family, is a widely utilized traditional medicinal herb in China. Historically, it has been employed to treat conditions such as influenza, nausea, anorexia, and various ailments associated with “pathogenic dampness”. To the best of our knowledge, this study presents the first systematic review of recent research on *E. fortunei*, based on a comprehensive literature search across both Chinese and international databases, including Web of Science, PubMed, SciFinder, and CNKI. The review encompasses its botanical characteristics, traditional applications, phytochemical composition, pharmacological properties, and toxicological profiles. Current research reveals a diverse array of phytochemicals in *E. fortunei*, with 162 compounds identified to date, including thymol derivatives, terpenoids, alkaloids, benzofurans, fatty acids, and other bioactive constituents. These compounds exhibit a broad spectrum of pharmacological activities, encompassing anti-cancer, anti-viral, anti-fungal, anti-inflammatory, and anti-diabetic effects. Among these, thymol derivatives and benzofurans emerge as the most prominent bioactive compounds, demonstrating potent cytotoxic effects against various tumor cell lines. Although *E. fortunei* is generally considered safe, certain pyrrolizidine alkaloids (PAs) present potential hepatotoxic risks, which can be mitigated through appropriate dosage control and formulation optimization. As a valuable traditional Chinese medicinal herb, *E. fortunei* exhibits substantial therapeutic potential. In conclusion, this review provides a comprehensive and systematic overview of current research on *E. fortunei*, offering scientific evidence and guidance for its rational development and clinical application.

## 1. Introduction

The genus *Eupatorium* (family Asteraceae) comprises approximately 30–60 species, predominantly distributed across the temperate and tropical regions of Central and South America, with limited representation on other continents [1,2,3]. Among the 14 *Eupatorium* species native to China, *Eupatorium fortunei* Turcz. (*E. fortunei*) is particularly notable for its dual significance as both an aromatic and medicinal plant, with a documented history of utilization spanning over two millennia [4]. This perennial herb is widely distributed throughout China’s subtropical and warm-temperate zones and also occurs in several other Asian countries, including Japan, Korea, Thailand, and Vietnam [5,6]. Since antiquity, *E. fortunei* has been an integral component of traditional medicine systems. It has been traditionally employed as a folk remedy for conditions such as edema, fever, chills, and nausea, and was attributed with properties to dispel dampness, alleviate summer heat, and promote overall health [7]. Notably, *E. fortunei* has been officially recognized by China’s National Health Commission as a medicinal plant authorized for use as a functional food ingredient [3].

*E. fortunei* contains a diverse array of phytochemical constituents. Various classes of compounds have been isolated and identified from its extracts, including terpenoids (predominantly monoterpene-derived thymol derivatives), flavonoids, phenylpropanoids, and pyrrolizidine alkaloids (PAs) [8]. Given its aromatic characteristics, previous phytochemical investigations have primarily focused on volatile oil components, particularly thymol derivatives, which are present in relatively high abundances. These compounds are regarded as the principal bioactive constituents of the plant and are considered essential for its broad-spectrum pharmacological activities [9,10]. Extensive pharmacological investigations have demonstrated that *E. fortunei* exhibits diverse biological activities, including anti-cancer, anti-viral, anti-fungal, anti-inflammatory, and anti-diabetic properties [11]. In the 2020 edition of the Pharmacopeia of the People’s Republic of China (ChP, 2020), volatile oil is designated as the primary quality control marker (Q-marker) for *E. fortunei*. As the plant’s predominant bioactive component, the volatile oil demonstrates substantial anti-inflammatory and anti-bacterial effects [12]. Currently, several traditional Chinese patent medicines and compound formulations containing *E. fortunei* are utilized primarily for treating digestive and respiratory disorders, as well as conditions associated with damp-heat syndromes. Additionally, during the coronavirus disease 2019 (COVID-19) pandemic in China, traditional Chinese medicine (TCM) formulations containing *E. fortunei* played a significant role in various stages of disease management [13].

Recent years have witnessed substantial progress in the isolation and identification of phytochemical constituents from *E. fortunei*, as well as in elucidating its pharmacological potential. Although modern pharmacological investigations have demonstrated its broad therapeutic effects, to the best of our knowledge, no systematic review has comprehensively summarized the research on this plant. This paper aims to address this gap by providing an integrated overview of recent advances in *E. fortunei* research, encompassing its botanical characteristics, traditional uses, phytochemistry, pharmacological activities, and toxicology. Additionally, it identifies current research gaps and limitations, offering critical insights into the plant’s research status and establishing a foundation for future investigations and applications.

## 2. Materials and Methods

A systematic literature search was conducted using databases including PubMed, Web of Science, SciFinder, ScienceDirect, and Google Scholar. The search keywords included “*Eupatorium fortunei*”, “*Eupatorium* species”, “phytochemistry”, “pharmacological activity”, and “toxicity”. The retrieved literature covered both representative classical studies and relevant research published in recent years, with the search period extending up to 2026. The included literature mainly comprised academic journal articles, monographs, and book chapters, while non-academic sources and duplicate publications were excluded. Furthermore, the reference lists of relevant publications were manually screened to identify additional studies that may have been missed during the database search.

## 3. Botany

### 3.1. Plant Sources

**Figure 1:**
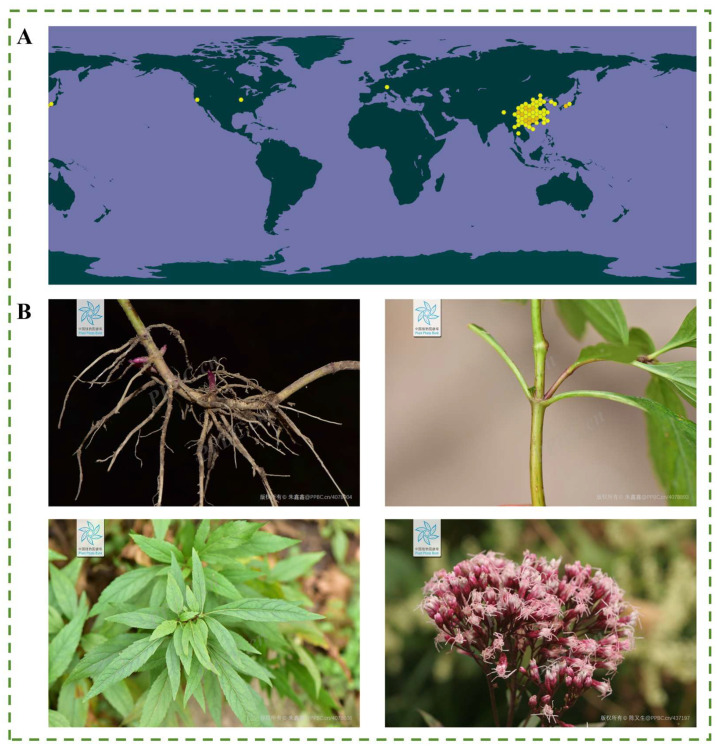
(**A**) The global geographical distribution of *Eupatorium fortunei* Turcz. (Source: www.gbif.org, accessed on 1 February 2026), and (**B**) the botanical illustration of *Eupatorium fortunei* Turcz. (The images were obtained from the Plant Photo Bank of China (PPBC), an open scientific resource. The watermarks on the images indicate the copyright holders and the source identification numbers).

*E. fortunei* is primarily distributed across several provinces in China, including Shandong, Jiangsu, Zhejiang, Jiangxi, Hubei, Hunan, Yunnan, Sichuan, Guizhou, Guangxi, Guangdong, and Shanxi. It also occurs in other Asian countries, such as Japan and Korea. The global geographical distribution of *E. fortunei* is documented in the GBIF online database (www.gbif.org), as illustrated in Figure 1A.

### 3.2. Taxonomy

*E. fortunei* is a perennial herb belonging to the genus *Eupatorium* of the family Asteraceae. According to authoritative international plant taxonomic databases, including WFO Plant List, Kew, and Flora of China, *Eupatorium fortunei* Turcz. is currently recognized as the accepted name for this taxon and is classified within the genus *Eupatorium*. Moreover, recent taxonomic studies have suggested a different classification. The Kew botanist specializing in the family Asteraceae proposed that *E. fortunei* should currently be treated as a synonym of *Eupatorium japonicum* Thunb., reflecting ongoing taxonomic uncertainty and debate regarding the classification and phylogenetic relationships within this group [14]. In recent years, advances in systematic taxonomy have further clarified species delimitation and phylogenetic relationships within the genus *Eupatorium*. However, in studies related to medicinal botany and ethnopharmacology, the traditional name *E. fortunei* continues to be widely used. Meanwhile, comparative analyses of chloroplast genome sequences and phylogenetic studies have provided reliable molecular evidence for clarifying the taxonomic position of *E. fortunei* within the genus *Eupatorium* and its phylogenetic relationships with related species, thereby offering important support for its taxonomic placement [15].

### 3.3. Plant Traits

*E. fortunei* typically thrives in warm and humid climates. According to the Flora of China (http://www.iplant.cn, accessed on 1 February 2026) [16], it typically attains a height of 40–100 cm. The plant possesses horizontal rhizomes that are pale reddish-brown. Its stems are erect, green or reddish-purple, exhibiting sparse branching and generally forming corymbs only at the apex. The stems and branches are sparsely covered with short, soft trichomes, whereas the inflorescence branches and peduncles exhibit denser pubescence. The mid-cauline leaves are relatively large, predominantly ternately lobed or deeply ternately divided. The central leaflet is oblong to lanceolate, while the lateral lobes are smaller but similar in morphology. The upper cauline leaves are typically entire, commonly lanceolate or oblong-lanceolate in form. The leaf surfaces are smooth and glabrous, lacking glandular punctae, and the pinnate venation is prominent. The leaf margins exhibit coarse or irregularly serrate dentition. The dimensions of the mid- and lower cauline leaves gradually diminish, with basal leaves frequently senescing during anthesis.

According to ChP, 2020, the microscopic characteristics of *E. fortunei* leaves are mainly as follows: the anticlinal walls of the upper epidermal cells are slightly curved, whereas those of the lower epidermal cells are sinuously curved. Non-glandular trichomes composed of several cells are present on the leaf surface, with longer trichomes occurring along the leaf veins. The stomata are of the anomocytic type [17].

The capitula of *E. fortunei* are numerous and borne at the stem apex or branch tips, arranged in compound corymbs. The involucre is campanulate, with the involucral bracts arranged in two to three imbricate layers; the outer bracts are shorter, whereas the middle and inner bracts are longer. The bracts are purplish-red, with smooth, glabrous surfaces lacking glandular dots, and have obtuse apices. The flowers are white or slightly pinkish, with tubular corollas. The fruit is an achene, dark brown, oblong in shape, and characterized by five longitudinal ribs, with a white pappus. The flowering and fruiting period generally occurs from July to November. Photographs of the roots, leaves, flowers, and fruits of *E. fortunei* are presented in Figure 1B.

## 4. Traditional Uses

As an aromatic plant with a long-standing history of medicinal use, *E. fortunei* has been integral to traditional medical systems in numerous countries. Ancient Chinese medical texts, including *Shen Nong’s Materia Medica* and *Compendium of Materia Medica*, document its use as a common herb in folk medicine for treating various disorders associated with pathogenic dampness. In China, its medicinal use dates back to the Western Han Dynasty, approximately two millennia ago. Notably, due to its pleasant fragrance, *E. fortunei* was traditionally worn by women and children on their clothing to ward off malodorous conditions and prevent diseases. This custom led to the herb being designated “Pei Lan”, meaning “the herb to be worn” [4,16]. In TCM, *E. fortunei* is characterized as pungent in taste and neutral in nature, with affinity for the spleen, stomach, and lung meridians. It is believed to resolve dampness, invigorate the spleen, promote appetite, and dispel summer heat [18]. TCM theory emphasizes the concepts of nature, flavor, and meridian tropism, which guide clinical application [19,20,21,22]. The herb’s nature, flavor, and meridian affinity determine its therapeutic orientation and pharmacodynamic properties within the body. According to these principles, *E. fortunei* is commonly employed to alleviate symptoms associated with dampness stagnation, including nausea, vomiting, and anorexia [23]. It is also regarded as a diuretic and detoxifying agent, frequently prescribed as an adjunct therapy for conditions such as chills, edema, and fever [24]. In clinical practice, *E. fortunei* can be administered alone or in combination with other TCM herbs, such as *Atractylodes lancea*, *Magnolia officinalis*, and *Agastache rugosa*, to enhance its therapeutic effects in eliminating dampness and turbidity, regulating qi, harmonizing the middle burner, and relieving exterior syndromes associated with summer heat [25,26,27,28]. According to Ch. P, 2020, *E. fortunei* is primarily indicated for conditions such as damp-turbid obstruction in the middle jiao, epigastric distension, nausea, a sweet or greasy taste in the mouth, halitosis, excessive salivation, exogenous summer-dampness syndrome, early-stage damp-heat disease, fever with fatigue, and chest oppression. The recommended clinical dosage ranges from 3 to 10 g [17].

Beyond China, *E. fortunei* plays a significant role in other Asian traditional medical systems. In Korean medicine, it is prescribed for gastrointestinal disorders associated with dampness stagnation, summer-heat colds, edema, and pediatric febrile convulsions. Its therapeutic effects align closely with those in TCM, focusing on resolving dampness through aromatic properties, strengthening the spleen, and harmonizing the middle burner. It is employed to alleviate symptoms such as poor appetite, nausea, diarrhea, dizziness, and edema, and is particularly efficacious in preventing and treating dampness-related conditions in hot and humid climates. In some folk remedies, *E. fortunei* is decocted and administered to treat pediatric summer-heat convulsions or fever and edema induced by damp-heat, reflecting its mild diuretic, anti-pyretic, and sedative properties [24]. In Vietnamese traditional medicine, it is commonly employed as a diuretic, anti-bacterial, and anti-pyretic agent for treating edema, ascites, and inflammatory diseases [29]. Across Chinese, Korean, and Vietnamese medical systems, the traditional uses of *E. fortunei* demonstrate remarkable consistency, with a shared emphasis on its ability to strengthen the spleen and stomach, eliminate dampness and turbidity, and clear summer heat. Modern pharmacological research has further validated its diverse biological activities, including anti-inflammatory, anti-fungal, and anti-viral effects. These findings not only corroborate its traditional applications but also underscore its potential as a valuable natural medicinal and functional plant resource.

## 5. Phytochemistry

**Figure 2:**
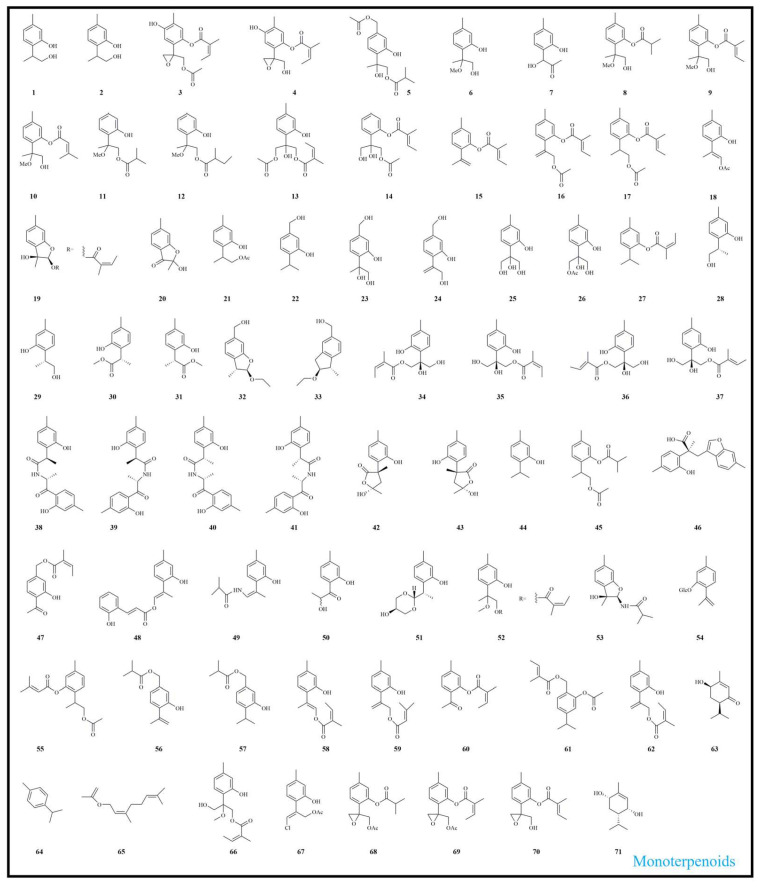
Structural characterization of monoterpenoids from *Eupatorium fortunei* Turcz.

**Figure 3:**
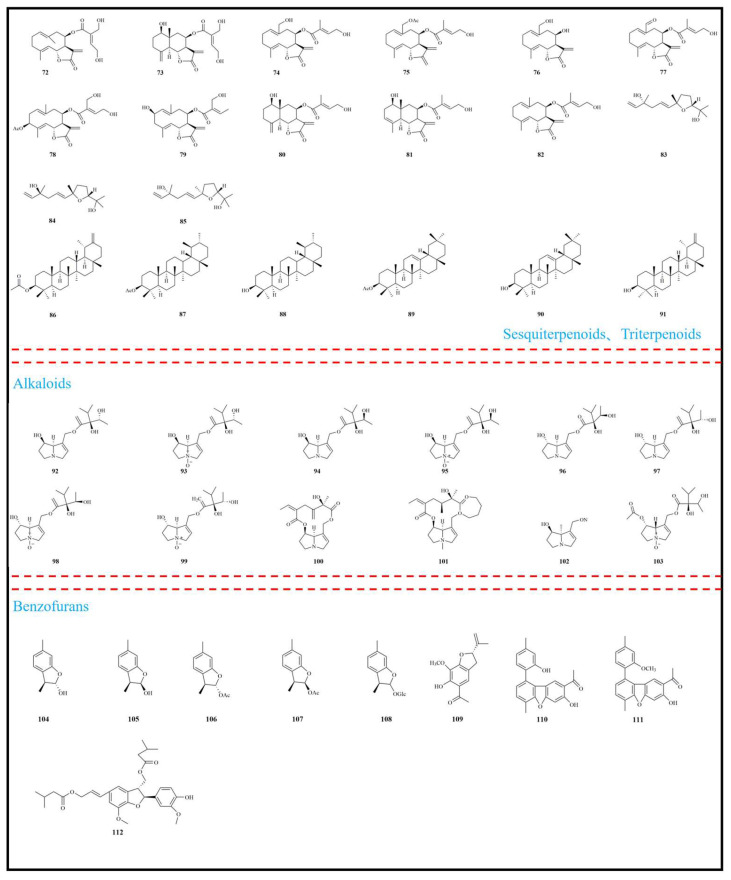
Structural characterization of sesquiterpenoids, triterpenoids, alkaloids, and benzofurans from *Eupatorium fortunei* Turcz.

**Figure 4:**
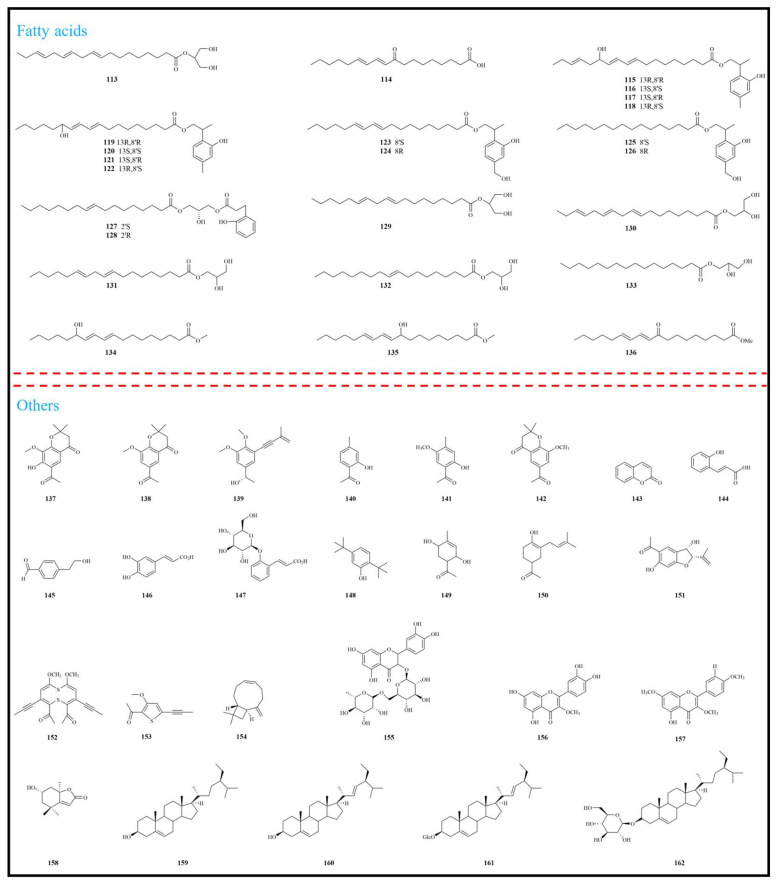
Structural characterization of fatty acids and other compounds from *Eupatorium fortunei* Turcz.

To date, 162 phytochemical constituents have been isolated and identified from *E. fortunei*, encompassing a broad spectrum of structural classes, including terpenoids, alkaloids, benzofurans, fatty acids, and other compounds. Among these, terpenoids and benzofurans represent the principal bioactive constituents, present in relatively high concentrations. Detailed information regarding these compounds is summarized in Table 1, with their chemical structures illustrated in Figure 2, Figure 3 and Figure 4.

### 5.1. Terpenoids

Terpenoids are among the most widely distributed and structurally diverse primary and secondary metabolites in nature. Composed of isoprene units, they exhibit diverse chemical structures and significant biological activities [39,40]. Terpenoids constitute one of the major classes of bioactive constituents in *E. fortunei* and have been widely reported from this plant. In recent years, researchers have isolated numerous novel terpenoids from *E. fortunei*. To date, a total of 91 terpenoids (**1**–**91**) have been isolated and identified from *E. fortunei*, of which compounds **1**–**71** are monoterpenoids, **72**–**85** are sesquiterpenoids, and **86**–**91** is a triterpene. Notably, thymol derivatives (**30**–**41**) were the six pairs of enantiomers first isolated from *E. fortunei*. Thymol is a natural monoterpene phenol (*p*-cymen-3-ol) and a structural isomer of carvacrol, and it has been reported to exhibit diverse biological activities [41,42]. Previous studies have demonstrated that thymol monoterpenoids represent the principal bioactive constituents of *E. fortunei* and exhibit diverse pharmacological activities, including anti-tumor, anti-inflammatory, and anti-diabetic effects [4,6,9,34]. The chemical structures of terpenoids isolated from *E. fortunei* are presented in Figure 2 and Figure 3.

### 5.2. Alkaloids

Among diverse secondary metabolites, alkaloids have garnered considerable attention owing to their distinctive nitrogen-containing heterocyclic structures and broad spectrum of biological activities [43,44]. These compounds are predominantly derived from plants, although they are also present in certain insects and animals [45]. PAs are the most widely distributed classes of plant-derived toxic compounds in nature and have attracted global attention due to their pronounced hepatotoxicity, genotoxicity, and embryotoxicity [46,47]. Using liquid chromatography–tandem mass spectrometry (LC-MS/MS), Zhang et al. (2021) identified and characterized eight PAs from the methanol extract of *E. fortunei*, namely intermedine (**92**), intermedine N-oxide (**93**), lycopsamine (**94**), lycopsamine N-oxide (**95**), rinderine (**96**), seneciphylline (**97**), senkirkine (**98**), and 7-acetylintermedine N-oxide (**99**), which collectively accounted for more than 93% of the total PA content in the plant [36]. Subsequently, additional PAs were isolated and identified from the 50% ethanol extract of *E. fortunei* in a 2022 study, including echinatine (**100**), rinderine N-oxide (**101**), echinatine N-oxide (**102**) and retronecine (**103**), among which echinatine N-oxide (**102**) was found to be the predominant component [37]. Notably, accumulating evidence has demonstrated that PAs present in *E. fortunei* can induce significant hepatotoxicity in vivo animal models through multiple mechanisms, including inflammation and oxidative stress, promotion of hepatic fibrosis, and hepatocellular apoptosis. Prolonged exposure may also result in neurological impairment [36]. Therefore, careful consideration must be given to the potential safety risks associated with the clinical application of *E. fortunei*. The chemical structures of alkaloids isolated from *E. fortunei* are illustrated in Figure 3.

### 5.3. Benzofurans

Among the biologically active heterocyclic compounds found in nature, benzofuran derivatives occupy a prominent position [48,49]. A limited number of benzofuran-type compounds (**104**–**112**) have been identified from the aerial parts of *E. fortunei*. As early as 2008, Jiang et al. first isolated three benzofuran derivatives from the methanol extract of *E. fortunei* [7], including 3*β*,6-dimethyl-2,3-dihydrobenzofuran-2*α*-ol (**104**) and 3*β*,6-dimethyl-2,3-dihydrobenzofuran-2*β*-ol (**105**), which are *C*-2 epimers, as well as 3*β*,6-dimethyl-2,3-dihydrobenzofuran-2*β*-*O*-*β*-D-glucopyranoside (**108**), the corresponding glycosidic derivative. Subsequently, two novel benzofuran derivatives, eupatodibenzofuran A (**110**) and eupatodibenzofuran B (**111**), were isolated and identified from *E. fortunei*. These compounds possess a unique dibenzofuran skeleton characterized by the structural motif 1-(9-(4-methylphenyl)-6-methyldibenzo[*b*,*d*]furan-2-yl) ethanone. Further studies revealed that both compounds could induce apoptosis in A549 and MCF-7 cells, exhibiting significant anti-tumor activity [30]. The chemical structures of benzofurans isolated from *E. fortunei* are illustrated in Figure 3.

### 5.4. Fatty Acids

Fatty acids were predominantly isolated from the 95% ethanol extract of *E. fortunei*. To date, a total of 24 fatty acid compounds (**113**–**136**) have been isolated and characterized from *E. fortunei*, most of which possess long-chain polyhydroxy or keto-carboxyl substituted structures and represent typical polyoxygenated fatty acid natural products. Representative compounds include eupatorid A-G (each existing as two enantiomers), which exhibit significant anti-inflammatory activity in vitro. Notably, *E. fortunei* holds potential as a functional food additive, and its fatty acid constituents represent an important direction and prospective research focus for future investigations [50,51]. The structures of compounds **113**–**136** are summarized in Figure 4.

### 5.5. Others

In addition to the aforementioned compounds, approximately 26 additional constituents have been isolated from *E. fortunei*. As illustrated in Figure 4, these compounds primarily include phenethyl ketones (**137**–**141**), chromones (**142**), coumarins (**143**), phenolic acids (**144**–**147**), acetophenones (**149**–**151**), and sterols (**159**–**162**). Moreover, 6-acetyl-8-methoxy-2,2-dimethylchroman-4-one (**142**) represents a typical 4-chromanone derivative characterized by an aromatic ketone structure bearing a carbonyl group and was identified as the first compound possessing a 4-chromanone skeleton isolated from *E. fortunei*.

## 6. Pharmacology

Extensive research has documented the pharmacological properties of *E. fortunei*. Investigations of its various extracts and bioactive compounds have revealed significant therapeutic effects, including anti-cancer, anti-viral, anti-fungal, anti-inflammatory, and anti-diabetic activities. To date, pharmacological studies have predominantly focused on extracts derived from the aerial parts of the plant and their bioactive constituents. Table 2 summarizes the diverse pharmacological activities of *E. fortunei*.

### 6.1. Anti-Cancer Effect

Cancer remains one of the leading causes of mortality worldwide, contributing to millions of deaths annually and posing a substantial threat to public health. Efforts to combat this disease continue to intensify [55,56]. Recent pharmacological studies have demonstrated that the aqueous extract of *E. fortunei* significantly suppresses tumor metastasis and angiogenesis. A study employed B16F10 melanoma and HT1080 fibrosarcoma cell models to systematically investigate the underlying mechanisms through which the aqueous extract of *E. fortunei* modulates tumor metastasis and angiogenesis. The results revealed that, at non-cytotoxic concentrations (25–100 μg/mL), the extract markedly inhibited anchorage-independent colony formation, migration, and invasion of tumor cells. These effects were closely associated with the downregulation of MMP-9 mRNA expression and proteolytic activity, mediated through the suppression of p38/JNK phosphorylation and NF-κB activation. With respect to angiogenesis, the extract reduced VEGF mRNA and protein expression, blocked HIF-1*α* accumulation, and inhibited the Akt/mTOR signaling pathway, thereby suppressing human umbilical vein endothelial cell (HUVEC) migration, tube formation, and microvessel sprouting from rat aortic rings. In vivo experiments, daily oral administration of the *E. fortunei* aqueous extract at 50 mg/kg significantly reduced the number of pulmonary metastatic nodules in C57BL/6 mice without inducing apparent systemic toxicity. Notably, the extract inhibited MMP-9 activity and VEGF production through dual mechanisms, thus concurrently suppressing both tumor metastasis and angiogenesis. These findings suggest that *E. fortunei* extract may serve as a safe and effective natural anti-cancer agent with substantial therapeutic potential [24].

**Figure 5:**
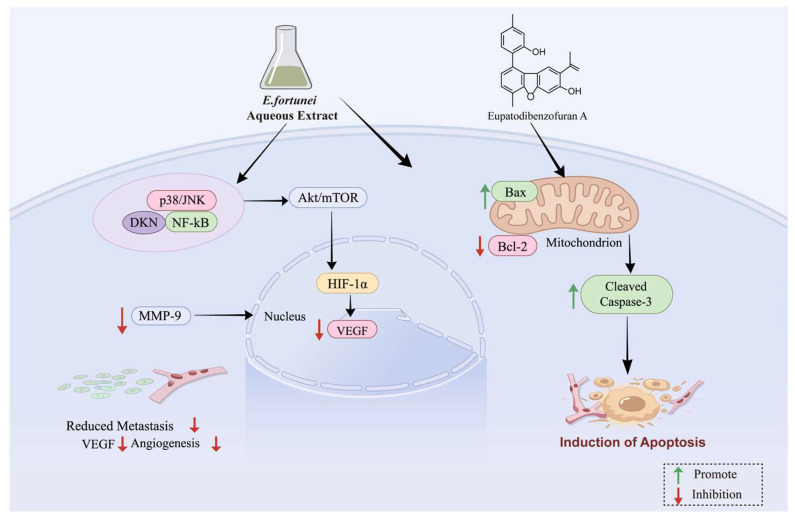
Potential mechanisms of the anti-cancer effect of *Eupatorium fortunei* Turcz.

Extensive research has identified numerous natural compounds as potential therapeutic agents for cancer [57,58]. Various chemical constituents of *E. fortunei* exhibit inhibitory effects against multiple cancer cell types, underscoring its promising potential as an anti-tumor agent. Modern studies have demonstrated that the bioactive constituents of *E. fortunei* can effectively suppress the growth of various malignancies, including breast, cervical, lung, liver, and colorectal cancers [30,32,35]. The mechanisms underlying these effects involve suppression of cell proliferation, induction of apoptosis, inhibition of telomerase activity, and blockade of tumor cell invasion and migration. From the 95% ethanol extract of the aerial parts of *E. fortunei*, four novel thymol derivatives were isolated: 9-angeloyloxy-8,9-dehydrothymol (**58**), 9-(3-methyl-2-butenoyloxy)-8,10-dehydrothymol (**59**), 7-isobutyryloxythymol (**57**), and 7-isobutyryloxy-8,9-dehydrothymol (**56**). Additionally, a novel isothymol derivative, 2-acetyl-7-tigloyloxy-isothymol (**61**), and one known analog, 9-*O*-angeloyl-8,10-dehydrothymol (**62**), were also identified. The structures of these compounds were elucidated using spectroscopic techniques, including infrared (IR) spectroscopy, ultraviolet (UV) spectroscopy, high-resolution electrospray ionization mass spectrometry (HR-ESI-MS), and nuclear magnetic resonance (NMR). Their cytotoxicity was evaluated against four human cancer cell lines (MCF-7, HeLa, A549, and HepG2) using the MTT assay. Compounds **58**, **59**, and **62** exhibited significant antiproliferative activity across all tested cell lines, with IC_50_ values ranging from 6.24 to 11.96 μM, comparable to those of the positive control cisplatin. In contrast, compounds **56**, **57**, and **61** showed weak activity (IC_50_ > 50 μM). A preliminary structure–activity relationship analysis suggested that the presence of a methyl group at *C*-1 and an acyl substituent at *C*-9 are critical determinants of cytotoxic activity [32]. These findings indicate that thymol derivatives from *E. fortunei* represent potential anti-cancer agents, providing a foundation for further pharmacological development and mechanistic investigations of this traditional medicinal herb. Additional phytochemical studies have identified several cytotoxic compounds from *E. fortunei*, with dibenzofuran derivatives being particularly noteworthy. For instance, Chang et al. (2021) successfully isolated and characterized a natural dibenzofuran compound, eupatodibenzofuran A (**110**), from the methanol extract of *E. fortunei*, demonstrating its potent anti-cancer activity [30]. This compound exhibited strong inhibitory effects on A549 cells and MCF-7 cells, with IC_50_ values of 5.95 ± 0.89 μM and 5.55 ± 0.23 μM, respectively, significantly exceeding the activity of the positive control 5-fluorouracil (10.57 ± 1.89 μM and 8.59 ± 1.03 μM). Mechanistic studies revealed that its cytotoxic effects were mediated through mitochondrial-dependent apoptosis, as evidenced by upregulation of Bax and cleaved caspase-3 expression, coupled with downregulation of B-cell lymphoma-2 (Bcl-2) levels. The proposed anti-cancer mechanism is illustrated in Figure 5. Additionally, other compounds from *E. fortunei*, such as eupatodithiecine, have exhibited moderate cytotoxicity, suggesting that the diverse secondary metabolites in this herb may exert synergistic anti-cancer effects.

In another study, Lee et al. (2020) utilized NMR-based metabolomics in conjunction with the SMART 2.0 compound annotation tool to examine the cytotoxic effects of representative sesquiterpene lactones from *E. fortunei* on several human cancer cell lines, including A549, MCF-7, SKOV3, HEp-2, and PC3 cells [35]. The results demonstrated that several compounds exhibited cytotoxic activity, with compound **80** displaying the lowest IC_50_ value (3.9 ± 0.6 μM) in PC3 cells, while compound **79** showed the strongest activity in MCF-7 cells (IC_50_ = 5.8 ± 0.1 μM). Furthermore, several bioactive constituents displayed broad-spectrum cytotoxicity across all five tested cancer cell lines. Notably, recent studies on *Eupatorium japonicum*, a synonym of *E. fortunei*, have further confirmed the anti-cancer potential of this class of compounds. Phan et al. (2021) isolated the sesquiterpene lactone eupatoriopicrin (**72**) from this plant, which exhibited significant cytotoxic activity against HepG2, MCF-7, and NTERA-2 cancer stem cells, with IC_50_ values of 0.94 ± 0.12, 1.22 ± 0.10, and 0.88 ± 0.05 μg/mL, respectively [34]. Mechanistic studies indicated that eupatoriopicrin (**72**) suppresses cancer cell proliferation by inducing caspase-3–dependent apoptosis. Taken together, current evidence suggests that *E. fortunei* and its bioactive constituents can regulate multiple key biological processes involved in tumor progression, including cell proliferation, apoptosis, metastasis, and angiogenesis—through multi-target mechanisms, highlighting their considerable potential as natural anti-cancer agents.

### 6.2. Anti-Viral Effect

**Figure 6:**
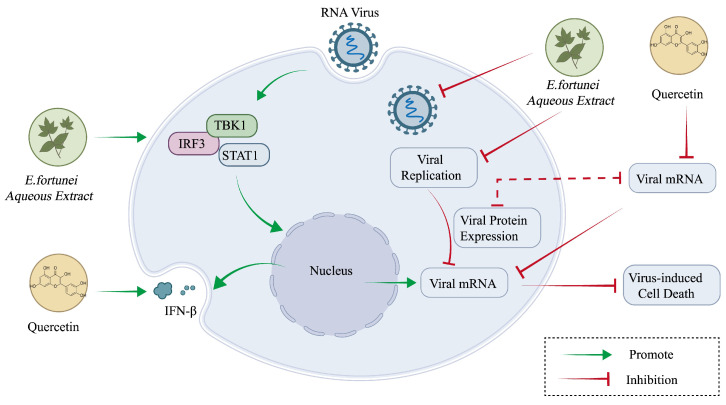
Potential mechanisms of the anti-viral effect of *Eupatorium fortunei* Turcz.

Viral diseases pose a significant global health threat, necessitating the urgent development of effective anti-viral agents [59,60]. Modern pharmacological and clinical studies have demonstrated that TCM possesses anti-viral activity and can inhibit a variety of viruses [61]. *E. fortunei* is among these, with an early study suggesting its anti-viral potential [10]. Reports indicate that the aqueous extract of *E. fortunei* may enhance the innate immune response against RNA virus infections by significantly inducing the secretion of interferon-*β* (IFN-*β*) and inflammatory cytokines such as tumor necrosis factor-*α* (TNF-*α*) and interleukin-6 (IL-6), as well as activating key signaling pathways such as interferon regulatory factor 3 (IRF3), signal transducer and activator of transcription 1 (STAT1), and TANK-binding kinase 1 (TBK1). In a study, GFP-labeled influenza virus H1N1, Newcastle disease virus (NDV), and vesicular stomatitis virus (VSV) were used to infect RAW 264.7 macrophages; subsequent to treatment with the aqueous extract of *E. fortunei*, viral replication and host cell responses were further evaluated. The results revealed that, at concentrations below 500 μg/mL, the aqueous extract significantly reduced viral fluorescent protein expression and replication while also inhibiting virus-induced cell death without exhibiting significant cytotoxicity. Additionally, ultra-performance liquid chromatography–tandem mass spectrometry (UPLC-MS/MS) analysis identified quercetin, psoralen, and quercitrin as the primary bioactive components, with quercetin exhibiting the strongest anti-viral activity. Quercetin significantly downregulated the mRNA expression levels of influenza virus genes, including Hemagglutinin (HA), Matrix Protein 2 (M2), Nucleoprotein (NP), Polymerase Acidic Protein (PA, PB2), and Nonstructural Protein 1 (NS-1), while promoting IFN-*β* secretion [52]. These findings suggest that *E. fortunei* exerts broad-spectrum anti-viral effects against RNA viruses through dual mechanisms involving immune modulation and direct viral inhibition, providing critical experimental evidence for its potential as a lead anti-viral compound. The schematic diagram of the anti-viral mechanism of *E. fortunei* is shown in Figure 6.

### 6.3. Anti-Fungal Effect

**Figure 7:**
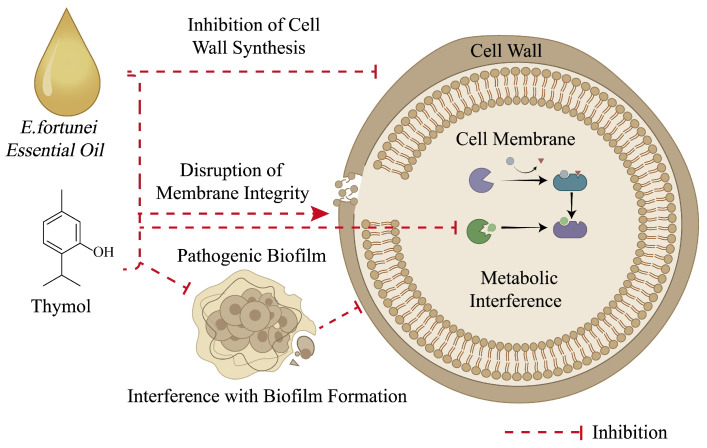
Potential mechanisms of the anti-fungal effect of the essential oil and thymol of *Eupatorium fortunei* Turcz.

The essential oil of *E. fortunei* has been reported to exhibit certain anti-fungal activity [6]. Previous studies have suggested that its mechanism of action may involve disruption of fungal cell membrane integrity, inhibition of cell wall synthesis, and interference with biofilm formation in pathogenic fungi [62]. A study published in 2020 systematically evaluated the inhibitory effects of the essential oil of *E. fortunei* on seven plant pathogenic fungi isolated from the rhizosphere of diseased *Panax notoginseng*. The results demonstrated that *E. fortunei* essential oil inhibited the mycelial growth of various fungal species at a concentration of 50 mg/mL, with minimum inhibitory concentration (MIC) values ranging from 0.20 to 1.17 mg/mL. Among its major constituents, thymol displayed strong anti-fungal activity, with MIC values ranging from 0.12 to 0.31 mg/mL, comparable to those of the positive control hymexazol (MIC = 0.05–0.63 mg/mL) [63]. Further in vivo experiments demonstrated that treatment with the essential oil of *E. fortunei* reduced the incidence of *Fusarium oxysporum* infection in *Panax notoginseng* seedlings to a certain extent and also promoted plant growth. Notably, when the essential oil of *E. fortunei* was combined with the chemical fungicide hymexazol, a certain synergistic inhibitory effect against *Cylindrocarpon destructans* was observed, suggesting its potential application in the control of plant diseases [12]. Taken together, current studies indicate that the essential oil of *E. fortunei* and its major constituents may inhibit fungal growth through multiple mechanisms of action. However, the available research remains limited, and the precise mechanisms underlying its anti-fungal activity, as well as its potential applications, require further systematic investigation. A schematic diagram illustrating the anti-fungal mechanisms of the essential oil constituents of *E. fortunei* is shown in Figure 7.

### 6.4. Anti-Inflammatory Effect

Inflammation is a key underlying factor in many chronic diseases, and the development of natural anti-inflammatory agents has garnered significant attention [64,65]. Plant-derived natural products, known for their chemical diversity, bioactivity, and relatively high safety profiles, are increasingly recognized as promising candidates for the prevention and treatment of inflammation [66]. Accumulating evidence suggests that various bioactive constituents of *E. fortunei*, including fatty acids, lignans, sesquiterpenes, and steroids, exhibit notable anti-inflammatory properties. 26 fatty acid derivatives, one lignan compound (brachangobinan A, BBA), and two monoterpenes were isolated from the 95% ethanol extract of the aerial parts of *E. fortunei*. An acute inflammatory model was established using Lipopolysaccharide (LPS)-induced RAW264.7 macrophages, and bioactive components were screened via an assay that inhibited NO production. The results revealed that BBA exhibited the strongest anti-inflammatory activity, significantly more potent than the fatty acid derivatives. Further network pharmacology analysis confirmed that BBA exerts its anti-inflammatory effects by inhibiting the NF-κB signaling pathway and downregulating the expression of pro-inflammatory cytokines, including TNF-*α*, IL-6, and Interleukin-1 *β* (IL-1*β*) [4]. Additionally, recent studies have demonstrated that newly isolated sesquiterpenes and steroidal compounds from the ethanol extract of *E. fortunei* also exhibit moderate NO inhibitory activity in the same inflammatory model (IC_50_ = 24.4–43.5 μM). These findings suggest that the anti-inflammatory effects of *E. fortunei* are attributable not only to its aromatic volatile components but also to various non-volatile secondary metabolites [11]. Further studies have revealed that the sesquiterpene lactone eupatoriopicrin (**72**) significantly inhibits NO production in LPS-induced RAW264.7 macrophages (IC_50_ = 7.53 ± 0.28 μg/mL). Moreover, it exerts anti-inflammatory effects by regulating inflammation-related signaling pathways, including TNF-α, IL-8, COX-2, and NF-κB [34].

### 6.5. Anti-Diabetic Effect

Diabetes mellitus is a global health issue characterized by elevated blood glucose levels. Among its types, type 2 diabetes mellitus (T2DM) is a prevalent chronic metabolic disorder affecting over 400 million people worldwide [67,68]. It results from a combination of genetic and environmental factors and is characterized by hyperglycemia and insulin resistance [69,70]. Recently, there has been increasing interest in the role of TCM in the prevention and treatment of diabetes [71]. *E. fortunei* clears heat, resolves dampness, invigorates the spleen, and relieves summer heat according to TCM theory, offering potential benefits in the prevention and management of metabolic diseases, particularly T2DM. Network pharmacology and molecular docking analyses have demonstrated that *E. fortunei* regulates glucose and lipid metabolic disorders through multiple targets and pathways. Core target prediction identified AKT serine/threonine kinase 1 (AKT1), insulin (INS), IL-6, TNF, peroxisome proliferator-activated receptor gamma (PPARG), and mitogen-activated protein kinase 8 (MAPK8) as key nodes within the pharmacological network, suggesting that *E. fortunei* may exert hypoglycemic effects by enhancing insulin signaling, modulating inflammatory responses, and regulating lipid metabolism. Furthermore, KEGG pathway enrichment analysis revealed that the primary regulatory pathways involve phosphatidylinositol 3-kinase-AKT signaling pathway (PI3K-Akt), hypoxia-inducible factor 1 (HIF-1), TNF, and MAPK signaling, all of which are closely associated with insulin resistance, inflammatory cytokine release, and energy metabolism regulation. Molecular docking studies confirmed strong binding affinities between major constituents and target proteins such as AKT1, PPARG, and IL-6, supporting the hypothesis that compounds in *E. fortunei* may synergistically improve insulin sensitivity and alleviate insulin resistance [53].

Additionally, recent studies have demonstrated that thymol derivatives from *E. fortunei* exhibit inhibitory activity against *α*-glucosidase. The results demonstrated weak inhibitory effects within the concentration range of 1–256 μg/mL, with maximum inhibition rates of 5–14%. This suggests that their direct enzyme inhibitory activity is limited; however, they may indirectly enhance glucose metabolism through anti-oxidant and anti-inflammatory mechanisms [31]. In summary, *E. fortunei* appears to regulate glucose homeostasis through the synergistic actions of multiple constituents targeting key processes such as insulin signaling, lipid metabolism, and inflammatory responses. These findings underscore its potential as a natural hypoglycemic agent and as a promising candidate for developing metabolism-regulating functional foods.

### 6.6. Other Effects

In addition to the previously mentioned pharmacological activities, *E. fortunei* has been demonstrated in clinical and scientific studies to possess several other notable biological effects. The ethyl acetate-soluble extract from the aerial parts of *E. fortunei* was found to exhibit significant inhibitory activity against the growth of the freshwater cyanobacterium *Microcystis aeruginosa*. The bioactive constituents responsible for this effect were identified as polyhydroxylated thymol derivatives and phenolic acid derivatives. Among these, 8,9,10-trihydroxythymol demonstrated the strongest anti-cyanobacterial activity, achieving a 45.6% inhibition rate at a concentration of 50 μg/mL, comparable to that of the positive control copper sulfate (5 μg/mL, 47.5%), with an IC_50_ value of 62.4 ± 8.3 μg/mL [28]. These findings suggest that this compound from *E. fortunei* holds considerable ecological potential as a natural algicidal agent.

Furthermore, *E. fortunei* has exhibited significant natural anthelmintic activity in the prevention and control of parasitic infections. In an in vivo model using goldfish (*Carassius auratus*) infected with the monogenean parasite *Dactylogyrus intermedius*, methanol extracts from 26 traditional medicinal plants were subjected to a screening assay. The results revealed that treatment with *E. fortunei* extract at a concentration of 0.5 mg/mL for 48 h achieved a 100% deworming rate without causing any fish mortality, indicating excellent safety. Further solvent fractionation demonstrated that the chloroform extract of *E. fortunei* displayed the strongest anti-parasitic activity, outperforming the petroleum ether, ethyl acetate, methanol, and aqueous fractions. Compared to the control group, the treated group exhibited a significant reduction in parasite numbers in a concentration-dependent manner. The anthelmintic mechanism is hypothesized to involve polyphenolic and sesquiterpene secondary metabolites, which may disrupt the parasite’s surface structure, interfere with its energy metabolism, and inhibit its attachment capacity, thereby exerting potent anti-parasitic effects [54].

## 7. Toxicity

### 7.1. PA-Related Hepatotoxicity

Within the TCM system, certain “toxic medicinal herbs” with potent pharmacological effects hold irreplaceable clinical value [72,73,74]. These herbs often exhibit strong bioactivity, and when used according to the TCM principle of syndrome differentiation and treatment, their therapeutic benefits typically outweigh potential risks [75,76]. However, the potential toxicity of *E. fortunei* should not be overlooked. Ancient medical texts have already documented that prolonged use of *E. fortunei* may cause liver injury. Modern studies have confirmed that the toxicity of *E. fortunei* may be attributed to its content of PAs [77]. These compounds possess hepatotoxic, neurotoxic, and potentially carcinogenic properties and have been classified by the European Medicines Agency’s Committee on Herbal Medicinal Products (EMA/HMPC) as naturally occurring toxic constituents requiring strict regulatory control. The established maximum safe daily intake for adults is 1.0 μg [78,79,80]. Using LC-MS/MS analysis, Zhang et al. (2021) identified several PAs in *E. fortunei* and its related preparations, including intermedine, lycopsamine, and their N-oxides, retronecine, seneciphylline, senkirkine, and 7-acetylintermedine N-oxide, totaling eight major constituents [36]. The total PAs content ranged from 0.18 to 61.81 μg/g, with significant regional variation; samples collected from Jiangsu, Anhui, and Guangxi exhibited the highest concentrations. Risk assessments indicated that the estimated daily exposure levels of most samples exceeded the European safety limit by 60–600-fold, suggesting a potential risk of chronic liver injury with long-term or high-dose use. Further in vivo studies confirmed the hepatic accumulation of pyrrolic metabolites following oral administration. These reactive intermediates, likely generated through cytochrome P450 (CYP450)-mediated oxidation of PAs, were shown to induce hepatic veno-occlusive-like lesions, demonstrating characteristic features of PA-induced hepatotoxicity.

### 7.2. Potential Neurotoxicity of PAs

In addition to hepatotoxicity, the potential neurotoxicity of PAs warrants attention. Intermedine, lycopsamine, and their N-oxides present in *E. fortunei* extracts can cross the blood–brain barrier and act on neural progenitor cells (NPCs). In vitro experiments revealed that exposure to 10–30 μM intermedine N-oxide reduced NPC viability and inhibited their differentiation into oligodendrocytes. This effect was accompanied by a decrease in mitochondrial membrane potential, an increase in reactive oxygen species (ROS) levels, and upregulated expression of apoptosis-related proteins such as cleaved cysteine-dependent aspartate-specific protease-3 (caspase-3) and BCL2-associated X protein (Bax), indicating that oxidative stress and mitochondrial dysfunction play key roles in PA-induced neurotoxicity [81]. Furthermore, PAs from other sources have been shown to disrupt neuronal energy metabolism and induce apoptosis by inhibiting the Akt/mTOR signaling pathway [36].

### 7.3. Metabolomic Evidence of Hepatotoxicity

Metabolomics offers a comprehensive profile of the body’s metabolic state, aligning with the holistic principles of TCM, making it an essential tool for the scientific assessment of herbal toxicity [82,83,84,85]. Study finding, it was reported that continuous oral administration of the total alkaloid extract of *E. fortunei* at a dose of 25 mg/kg for four weeks induced significant hepatic injury in mice; this was evidenced by decreased body and liver weights, elevated serum aspartate aminotransferase (AST) and alanine aminotransferase (ALT) levels, and the occurrence of hepatic fibrosis. Metabolomic analysis revealed that the extract primarily affected the hepatic glycerophospholipid, glutathione, and bile acid metabolic pathways. Notably, dysregulation of glycerophospholipid metabolism-characterized by upregulation of lysophosphatidylglycerol acyltransferase 1 (LPGAT1) and downregulation of choline kinase beta (CHKB), choline/ethanolamine phosphotransferase 1 (CEPT1), and phospholipase D family member 4 (PLD4), was identified as a key event leading to inflammation and apoptosis. Concurrently, serum levels of IL-6, TNF-*α*, NF-κB, and inducible nitric oxide synthase (iNOS) were markedly increased, accompanied by decreased superoxide dismutase (SOD) activity and an elevated oxidized glutathione/reduced glutathione (GSSG/GSH) ratio, suggesting that oxidative stress and inflammatory responses may play important roles in the hepatotoxicity of *E. fortunei* [37].

### 7.4. Cytotoxic Secondary Metabolites

**Figure 8:**
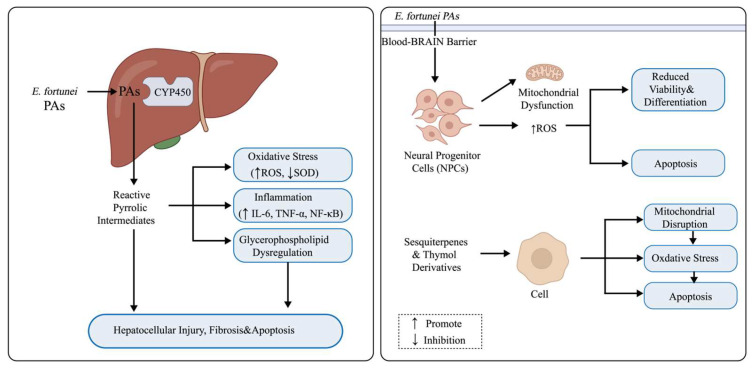
Potential mechanisms of the hepatotoxicity, neurotoxicity, and cytotoxicity of *Eupatorium fortunei* Turcz.

In addition to PAs, *E. fortunei* contains several secondary metabolites that exhibit cytotoxic activity in vitro, including sesquiterpene lactones, thymol derivatives, and dibenzofuran compounds [30,32,35]. Studies have demonstrated that sesquiterpene lactones isolated from *E. fortunei* exhibit potent inhibitory effects on breast, prostate, and lung cancer cells. These compounds can induce cytotoxic responses by triggering apoptosis, disrupting mitochondrial function, and activating oxidative stress pathways. The toxicological profile of *E. fortunei* appears to be complex, involving PA-related hepatotoxic risks and the presence of bioactive secondary metabolites. Consequently, future pharmaceutical and functional food development should prioritize rigorous quality control of raw material sources and PA content, employing integrated multi-omics approaches to define safe dosage ranges and toxicological thresholds, thereby providing a scientific foundation for the rational clinical application and risk management of *E. fortunei* [86,87]. The schematic diagrams of the hepatotoxicity, neurotoxicity, and cytotoxicity of *E. fortunei* are shown in Figure 8.

## 8. Conclusions and Future Perspectives

*E. fortunei*, an aromatic medicinal plant with significant therapeutic value, occupies a unique position within traditional medical systems. Current studies indicate that this plant contains a rich diversity of secondary metabolites, mainly including terpenoids, alkaloids, benzofurans, and fatty acids. These constituents are considered to constitute the key material basis underlying its various pharmacological activities, such as anti-cancer, anti-viral, anti-inflammatory, anti-fungal, and anti-diabetic effects. However, alongside its potential therapeutic value, non-negligible toxicological risks must also be considered. Several PAs identified in *E. fortunei* are regarded as the primary sources of its toxicity and may induce significant adverse effects, including hepatotoxicity, genotoxicity, and neurodevelopmental toxicity. Consequently, careful attention must be given to safety evaluations and dosage control during clinical applications and drug development to minimize potential adverse effects.

Although notable progress has been made in research on *E. fortunei* in recent years, the current body of evidence still presents several limitations, which to some extent hinder its further translation into evidence-based medicine and clinical practice. Future studies should therefore focus on several clearly defined directions. First, comprehensive phytochemical investigations of *E. fortunei* should be conducted using high-resolution analytical platforms to further update and expand the existing metabolite profiles, thereby providing a reliable chemical basis for subsequent pharmacological studies. Second, it is necessary to conduct systematic evaluations of pharmacokinetics, bioavailability, and toxicology to rigorously validate the reported biological activities of *E. fortunei*, rather than relying solely on in vitro experiments or in vivo animal models. Third, multidisciplinary approaches integrating metabolomics, network pharmacology, and artificial intelligence–based data mining should be employed to systematically elucidate the synergistic mechanisms underlying compound formulations or combination therapies of *E. fortunei*. Such strategies may help reveal its characteristic “multi-component, multi-target, and multi-pathway” mode of action and provide new perspectives for its rational development and utilization. Finally, more standardized and systematic clinical investigations should be conducted, accompanied by the establishment of comprehensive quality control and evaluation standards to promote the safe, standardized, and evidence-based application of *E. fortunei*. Systematically advancing and coordinating these research priorities will be crucial for scientifically validating its traditional therapeutic value and fully realizing its potential applications within modern evidence-based medicine.

## Figures and Tables

**Table 1:** Identification of compounds from *Eupatorium fortunei* Turcz.

No.	Compound Name	Molecular Formula	Extraction Solvent	Identification Method	Ref.
Monoterpenoids					
1	(8*S*)-9-hydroxythymol	C_10_H_14_O_2_	95% EtOH	ESIMS, NMR	[4]
2	(8*R*)-9-hydroxythymol	C_10_H_14_O_2_	95% EtOH	ESIMS, NMR	[4]
3	9-(acetyloxy)-8,10-epoxy-6-hydroxythymol-3-yl angelate	C_17_H_20_O_6_	MeOH	IR, HRCIMS, NMR	[6,9]
4	8,10-epoxy-9-hydroxythymol-3-yl tiglate	C_15_H_18_O_5_	MeOH	IR, HRCIMS, NMR	[6,9]
5	7-(acetyloxy)-8-hydroxy-9-[(2-methylpropanoyl) oxy] thymol	C_16_H_22_O_6_	MeOH	IR, HRCIMS, NMR	[6,9]
6	9-hydroxy-8-methoxythymol	C_11_H_16_O_3_	MeOH	IR, HRCIMS, NMR	[6,9]
7	1-hydroxy-1-(2-hydroxy-4-methylphenyl) propan-2-one	C_10_H_12_O_3_	MeOH	IR, HRCIMS, NMR	[6,9]
8	9-hydroxy-8-methoxythymol-3-yl (2-methylpropanoate)	C_15_H_22_O_4_	MeOH	IR, HRCIMS, NMR	[6,9]
9	9-hydroxy-8-methoxythymol-3-yl tiglate	C_16_H_22_O_4_	MeOH	IR, HREIMS, NMR	[6,9]
10	9-hydroxy-8-methoxythymol-3-yl (3-methylbut-2-enoate)	C_16_H_22_O_4_	MeOH	IR, HRCIMS, NMR	[6,9]
11	8-methoxy-9-[(2-methylpropanoyl) oxy] thymol	C_15_H_22_O_4_	MeOH	HREIMS, NMR	[6,9]
12	8-methoxy-9-[(2-methybutanoyl) oxy] thymol	C_16_H_24_O_4_	MeOH	HREIMS, NMR	[6,9]
13	10-(acetyloxy)-9-(angeloyloxy)-8-hydroxythymol	C_17_H_22_O_6_	MeOH	HREIMS, NMR	[6,9]
14	9-(acetyloxy)-8,10-dihydroxythymol-3-yl tiglate	C_17_H_22_O_6_	MeOH	HREIMS, NMR	[6,9]
15	8,9-dehydrothymol-3-yl tiglate	C_15_H_18_O_2_	MeOH	HREIMS, NMR	[6,9]
16	9-(acetyloxy)-8,10-dehydrothymol-3-yl tiglate	C_17_H_20_O_4_	MeOH	HREIMS, NMR	[6,9]
17	9-(acetyloxy) thymol-3-yl tiglate	C_17_H_22_O_4_	MeOH	HREIMS, NMR	[6,9]
18	(*E*)-2-(2-hydroxy-4-methylpheny1)-prop-l-en-1-yl acetate	C_12_H_14_O_3_	95% EtOH	MS, NMR	[3]
19	eupatobenzofuran	C_15_H_18_O_4_	95% EtOH	MS, NMR	[3]
20	2-hydroxy-2,6-dimethylbenzofuran-3 (2H)-one	C_10_H_10_O_3_	95% EtOH	MS, NMR	[3]
21	9-acetoxythymol	C_12_H_16_O_3_	95% EtOH	MS, NMR	[3]
22	7-hydroxythymol	C_10_H_14_O_2_	95% EtOH	MS, NMR	[3]
23	7,8,9-trihydroxythymol	C_10_H_14_O_4_	Organic, Aqueous	MS, NMR	[29]
24	8,10-didehydro-7,9-dihydroxythymol	C_10_H_12_O_3_	Organic, Aqueous	MS, NMR	[29]
25	8,9,10-trihydroxythymol	C_10_H_14_O_4_	Organic, Aqueous	MS, NMR	[29]
26	10-acetoxy-8,9-dihydroxythymol	C_12_H_16_O_5_	Organic, Aqueous	MS, NMR	[29]
27	thymyl angelate	C_15_H_20_O_2_	MeOH	UV, IR, MS, NMR	[30]
28	(+)-9-hydroxythymol	C_10_H_14_O_2_	95% EtOH	HRESIMS, NMR, HPLC	[3]
29	(−)-9-hydroxythymol	C_10_H_14_O_2_	95% EtOH	HRESIMS, NMR, HPLC	[3]
30	(+)-methyl 2-(2-hydroxy-4-methylphenyl) propanoate	C_11_H_14_O_3_	95% EtOH	HRESIMS, NMR, HPLC	[3]
31	(−)-methyl 2-(2-hydroxy-4-methylphenyl) propanoate	C_11_H_14_O_3_	95% EtOH	HRESIMS, NMR, HPLC	[3]
32	(+)-eupafortin B	C_12_H_16_O_2_	95% EtOH	UV, ESIMS, NMR	[3]
33	(−)-eupafortin B	C_12_H_16_O_2_	95% EtOH	UV, ESIMS, NMR	[3]
34	(+)-9-angeloyloxy-8,10-dihydroxythymol	C_15_H_20_O_5_	95% EtOH	HRESIMS, NMR, HPLC	[3]
35	(−)-9-angeloyloxy-8,10-dihydroxythymol	C_15_H_20_O_5_	95% EtOH	HRESIMS, NMR, HPLC	[3]
36	(+)-eupafortunin F	C_15_H_20_O_5_	95% EtOH	HRESIMS, NMR, HPLC	[3,10]
37	(−)-eupafortunin F	C_15_H_20_O_5_	95% EtOH	HRESIMS, NMR, HPLC	[3,10]
38	(+)-eupafortunin A	C_20_H_23_NO_4_	95% EtOH	HRESIMS, NMR, HPLC	[3]
39	(−)-eupafortunin A	C_20_H_23_NO_4_	95% EtOH	HRESIMS, NMR, HPLC	[3]
40	(+)-eupafortunin B	C_20_H_23_NO_4_	95% EtOH	HRESIMS, NMR, HPLC	[3,10]
41	(−)-eupafortunin B	C_20_H_23_NO_4_	95% EtOH	HRESIMS, NMR, HPLC	[3,10]
42	(+)-eupafortunin K	C_13_H_16_O_4_	95% EtOH	HRESIMS, NMR	[3,10]
43	(−)-eupafortunin K	C_13_H_16_O_4_	95% EtOH	HRESIMS, NMR	[3,10]
44	thymol	C_10_H_14_O	Hexane	GC-MS/MS	[23]
45	9-acetoxyl-3-isobutyroylthymol	C_16_H_22_O_4_	MeOH	HRESIMS, NMR	[10,31]
46	2-(2-hydroxy-4-methylphenyl)-2-methyl-3-(5-methylbenzofuran-3-yl) propanoic acid	C_20_H_20_O_4_	MeOH	HRESIMS, NMR	[10,31]
47	eupafortunin J	C_14_H_16_O_4_	95% EtOH	HRESIMS, NMR	[3,10]
48	eupafortunin C	C_19_H_18_O_4_	95% EtOH	HRESIM, NMR	[3,10]
49	eupafortunin E	C_14_H_19_NO_2_	95% EtOH	HRESIMS, NMR	[3,10]
50	eupafortunin H	C_10_H_12_O_3_	95% EtOH	HRESIMS, NMR	[3,10]
51	eupafortunin G	C_13_H_18_O_4_	95% EtOH	HRESIMS, NMR	[3,10]
52	9-angeloyloxy-8-methoxythymol	C_16_H_22_O_4_	95% EtOH	HRESIMS, NMR	[3]
53	eupafortunin D	C_14_H_19_NO_3_	95% EtOH	HRESIMS, NMR	[3,10]
54	8,9-dehydrothymol-3-o-*β*-glucoside	C_16_H_22_O_6_	95% EtOH	HRESIMS, NMR	[8,10]
55	9-(acetyloxy) thymol-3-yl (3-methylbut-2-enoate)	C_17_H_22_O_4_	95% EtOH	HRESIMS, NMR, HPLC	[8,10]
56	7-isobutyryloxy-8,9-dehydrothymol	C_14_H_18_O_3_	95% EtOH	UV, IR, HRESIMS, NMR	[10,32]
57	7-isobutyryloxythymol	C_14_H_20_O_3_	95% EtOH	UV, IR, HRESIMS, NMR	[10,32]
58	9-angeloyloxy-8,9-dehydrothymol	C_15_H_18_O_3_	MeOH	UV, IR, NMR, MS	[10,32]
59	9-(3-Methyl-2-butenoyloxy)-8,10-dehydrothymol	C_15_H_18_O_3_	MeOH	UV, IR, NMR, MS	[10,32]
60	eupafortunin I	C_14_H_16_O_3_	MeOH	UV, IR, NMR, MS	[10]
61	2-acetyl-7-tigloyloxy-isothymol	C_17_H_22_O_4_	95% EtOH	UV, IR, HRESIMS, NMR	[10,32]
62	9-*O*-angeloyl-8,10-dehydrothymol	C_15_H_18_O_3_	MeOH	HRESIMS	[32]
63	(4*β*,6*β*)-6-hydroxypiperitone	C_10_H_16_O_2_	95% EtOH	HRESIMS, NMR	[3]
64	*p*-cymene	C_10_H_14_	Hexane	GC-MS/MS	[23]
65	neryl acetate	C_13_H_22_O	Hexane	GC-MS/MS	[23]
66	9-*O*-Angeloxy-10-hydroxy-8-methoxythymol	C_16_H_22_O_5_	MeOH	MS, NMR	[33]
67	10-acetoxy-9-chloro-8,9-dehydrothymol	C_12_H_13_ClO_3_	MeOH	MS, NMR	[33]
68	9-acetoxy-8,10-epoxythymol-3-*O*-isobutyrate	C_16_H_20_O_5_	MeOH	MS, NMR	[33]
69	9-acetoxy-8,10-epoxythymol-3-*O*-angelate	C_17_H_20_O_5_	MeOH	MS, NMR	[33]
70	8,10-epoxy-9-hydroxythymol-3-*O*-tiglate	C_15_H_18_O_4_	MeOH	MS, NMR	[33]
71	*p*-menth-1-ene-3,6-diol	C_10_H_18_O_2_	MeOH	IR, MS, NMR	[34]
Sesquiterpenoids					
72	eupatoriopicrin	C_20_H_26_O_6_	MeOH	IR, MS, NMR	[34]
73	1-hydroxy-8-(4,5-dihydroxytigloyloxy)eudesma-4(15),11(13)-dien-6,12-olide	C_20_H_26_O_7_	MeOH	IR, MS, NMR	[34]
74	14-hydroxy-8*β*-[4′-hydroxytigloyloxy]-costunolide	C_20_H_26_O_6_	MeOH	HREIMS, NMR	[35]
75	14-acetoxy-8*β*-[4′-hydroxytigloyloxy]-costunolide	C_22_H_28_O_7_	MeOH	HREIMS, NMR	[35]
76	14-acetoxy-8*β*-hydroxy-costunolide	C_15_H_20_O_4_	MeOH	HREIMS, NMR	[35]
77	8*β*-[4′-hydroxytigloyloxy]-14-oxo-costunolide	C_20_H_24_O_6_	MeOH	HREIMS, NMR	[35]
78	3*β*-acetoxy-8*β*-[4′,5′-dihydroxytigloyloxy]-costunolide	C_22_H_28_O_8_	MeOH	HREIMS, NMR	[35]
79	2*β*-hydroxy-8*β*-[5′-hydroxytigloyloxy]-costunolide	C_20_H_26_O_6_	MeOH	HREIMS, NMR	[35]
80	1*β*-hydroxy-8*β*-[4′-hydroxytigloyloxy]-*β*-cyclocostunolide	C_20_H_26_O_6_	MeOH	HREIMS, NMR	[35]
81	1*β*-hydroxy-8*β*-[4′-hydroxytigloyloxy]-*α*-cyclocostunolide	C_20_H_26_O_6_	MeOH	HREIMS, NMR	[35]
82	8*β*-[4′-hydroxytigloyloxy]-costunolide	C_20_H_26_O_5_	MeOH	HREIMS, NMR	[35]
83	(+)-schensianol A	C_15_H_26_O_3_	95% EtOH	HREIMS, NMR	[11]
84	(+)-3-epi-schensianol A	C_15_H_26_O_3_	95% EtOH	HREIMS, NMR	[11]
85	(+)-3-epi-negunfurol	C_15_H_26_O_3_	95% EtOH	HREIMS, NMR	[11]
Triterpenoids					
86	taraxasteryl acetate	C_32_H_52_O_2_	MeOH	MS, NMR	[33]
87	α-amyrin acetate	C_32_H_54_O_2_	MeOH	IR, MS, NMR	[34]
88	α-amyrin	C_30_H_52_O	MeOH	IR, MS, NMR	[34]
89	*β*-amyrin acetate	C_32_H_52_O_2_	MeOH	IR, MS, NMR	[34]
90	*β*-amyrin	C_30_H_50_O	MeOH	IR, MS, NMR	[34]
91	taraxasterol	C_30_H_50_O	MeOH	UV, IR, NMR, MS	[30]
Alkaloids					
92	intermedine	C_16_H_27_NO_4_	MeOH	LC–MS	[36]
93	intermedine N-oxide	C_16_H_27_NO_5_	MeOH	LC–MS	[36]
94	lycopsamine	C_16_H_27_NO_4_	MeOH	LC–MS	[36]
95	lycopsamine N-oxide	C_16_H_27_NO_5_	MeOH	LC–MS	[36]
96	rinderine	C_15_H_25_NO_5_	MeOH	LC–MS	[36]
97	seneciphylline	C_18_H_23_NO_5_	MeOH	LC–MS	[36]
98	senkirkine	C_23_H_36_NO_5_	MeOH	LC–MS	[36]
99	7-acetylintermedine N-oxide	C_17_H_27_NO_7_	MeOH	LC–MS	[36]
100	echinatine	C_16_H_27_NO_4_	50% EtOH	LC–MS	[37]
101	rinderine N-oxide	C_16_H_27_NO_5_	50% EtOH	LC–MS	[37]
102	echinatine N-oxide	C_16_H_27_NO_5_	50% EtOH	LC–MS	[37]
103	retronecine	C_9_H_14_N_2_O_2_	50% EtOH	LC–MS	[37]
Benzofurans					
104	3*β*, 6-dimethyl-2, 3-dihydrobenzofuran-2*α*-ol	C_10_H_12_O_2_	MeOH	IR, EIMS, NMR	[7]
105	3*β*, 6-dimethyl-2, 3-dihydrobenzofuran-2*β*-ol	C_10_H_12_O_2_	MeOH	IR, EIMS, NMR	[7]
106	3*β*, 6-dimethyl-2, 3-dihydrobenzofuran-2*α*-yl acetate	C_12_H_14_O_3_	MeOH	UV, IR, EIMS, NMR,	[7]
107	3*β*, 6-dimethyl-2, 3-dihydrobenzofuran-2*β*-yl acetate	C_12_H_14_O_3_	MeOH	UV, IR, HRESIMS, NMR	[7]
108	3*β*,6-dimethyl-2,3-dihydrobenzofuran 2*β*-*O*-*β*-D-glucopyranoside	C_16_H_22_O_7_	MeOH	UV, IR, HRESIMS, NMR	[7]
109	6-hydroxy-7-methoxy-2-isopropenyl-5-acetylcumaran	C_14_H_16_O_4_	MeOH	UV, IR, MS, NMR	[30]
110	eupatodibenzofuran A	C_22_H_18_O_4_	MeOH	HRESIMS, NMR	[30]
111	eupatodibenzofuran B	C_23_H_20_O_4_	MeOH	HRESIMS, NMR	[30]
112	brachangobinan A	C_30_H_38_O_8_	95% EtOH	ESIMS, NMR	[4]
Fatty acids					
113	2-monolinolenin	C_21_H_36_O_4_	95% EtOH	UV, MS, NMR	[4,38]
114	9-oxo-10*E*,12*E*-octadecadienoic acid	C_18_H_30_O_3_	95% EtOH	UV, MS, NMR	[4]
115	(13R, 8′R)-eupatorid A	C_28_H_42_O_4_	95% EtOH	ESIMS, NMR	[4,10]
116	(13S, 8′S)-eupatorid A	C_28_H_42_O_4_	95% EtOH	ESIMS, NMR	[4,10]
117	(13S, 8′R)-eupatorid B	C_28_H_42_O_4_	95% EtOH	ESIMS, NMR	[4,10]
118	(13R, 8′S)-eupatorid B	C_28_H_42_O_4_	95% EtOH	ESIMS, NMR	[4,10]
119	(13R, 8′R)-eupatorid C	C_28_H_44_O_4_	95% EtOH	ESIMS, NMR	[4,10]
120	(13S, 8′S)-eupatorid C	C_28_H_44_O_4_	95% EtOH	ESIMS, NMR	[4,10]
121	(13S, 8′R)-eupatorid D	C_28_H_44_O_4_	95% EtOH	ESIMS, NMR	[4,10]
122	(13R, 8′S)-eupatorid D	C_28_H_44_O_4_	95% EtOH	ESIMS, NMR	[4,10]
123	(8′S)-eupatorid E	C_28_H_44_O_4_	95% EtOH	ESIMS, NMR	[4,10]
124	(8′R)-eupatorid E	C_28_H_44_O_4_	95% EtOH	ESIMS, NMR	[4,10]
125	(8′S)-eupatorid F	C_26_H_44_O_4_	95% EtOH	ESIMS, NMR	[4,10]
126	(8′R)-eeupatorid F	C_26_H_44_O_4_	95% EtOH	ESIMS, NMR	[4,10]
127	(2′S)-eupatorid G	C_29_H_46_O_6_	95% EtOH	ESIMS, NMR	[4,10]
128	(2′R)-eupatorid G	C_29_H_46_O_6_	95% EtOH	ESIMS, NMR	[4,10]
129	2-monolinolein	C_21_H_38_O_4_	95% EtOH	ESIMS, NMR	[4]
130	1-monolinolenoyl-rac-glycerol	C_21_H_36_O_4_	95% EtOH	ESIMS, NMR	[4]
131	1-monolinolein	C_21_H_38_O_4_	95% EtOH	ESIMS, NMR	[4]
132	1-oleoylglycerol	C_21_H_40_O_4_	95% EtOH	ESIMS, NMR	[4]
133	1-monopalmitin	C_19_H_38_O_4_	95% EtOH	ESIMS, NMR	[4]
134	13-hydroxy-9*E*,11*E*-octadecadienoic acid methyl ester	C_19_H_34_O_3_	95% EtOH	ESIMS, NMR	[4]
135	9-hydroxy-10*E*,12*E*-octadecadienoic acid methyl ester	C_19_H_34_O_3_	95% EtOH	ESIMS, NMR	[4]
136	methyl (10*E*,12*E*)-9-oxo-10,12-octadecadienoate	C_19_H_32_O_3_	95% EtOH	ESIMS, NMR	[4]
Others					
137	eupafortunin L	C_14_H_16_O_5_	95% EtOH	HRESIMS, NMR	[3,10]
138	eupafortunin M	C_14_H_16_O_4_	95% EtOH	HRESIMS, NMR	[3,10]
139	eupafortunin N	C_15_H_18_O_3_	95% EtOH	HRESIMS, NMR	[3,10]
140	2-hydroxy-4-methylacetophenone	C_9_H_10_O_2_	MeOH	UV, IR, MS, NMR	[30]
141	1-(2-hydroxy-5-methoxy-4-methylphenyl) ethanone	C_10_H_12_O_3_	MeOH	UV, IR, MS, NMR	[30]
142	6-acetyl-8-methoxy-2,2-dimethylchroman-4-one	C_14_H_16_O_4_	MeOH	HRESIMS, NMR	[30]
143	Coumarin	C_9_H_6_O_2_	MeOH	UV, IR, NMR, MS	[30]
144	trans-*o*-coumaric acid	C_9_H_8_O_3_	MeOH	UV, IR, MS, NMR	[30]
145	*O*-coumaric acid	C_9_H_10_O_2_	Organic, Aqueous	MS, NMR	[28]
146	caffeic acid	C_9_H_8_O_4_	MeOH	IR, MS, NMR	[34]
147	(2E)-3-[2-(*β*-D-glucopyranosyloxy)phenyl]-prop-2-en-oic acid	C_15_H_18_O_8_	MeOH	IR, MS, NMR	[34]
148	2,4-di-tert butylphenol	C_14_H_22_O	MeOH	UV, IR, MS, NMR	[30]
149	2,5-dihydroxy-4-methylacetophenone	C_9_H_14_O_3_	95% EtOH	MS, NMR	[3]
150	4-hydroxy-3-prenylacetophenone	C_13_H_20_O_2_	95% EtOH	MS, NMR	[3]
151	(2*S*,3*S*)-3,6-dihydroxytremetone	C_13_H_14_O_4_	95% EtOH	MS, NMR	[3]
152	1,1′-((2*E*,4*Z*,7*Z*,9*E*)-5,7-dimethoxy-3,9-di (prop-1-yn-1-yl)-1,6-dithiecine-2,10-diyl) diethanone	C_20_H_20_O_4_S_2_	MeOH	IR, HREIMS, NMR	[30]
153	2-acetyl-3-metoxy-5-(prop-1-ynyl)-thiophen	C_10_H_10_O_2_S	95% EtOH	HRESIMS, NMR	[3]
154	*β*-caryophyllene	C_14_H_22_	Hexane	GC-MS/MS	[23]
155	quercetin-3-*O*-rutinoside	C_27_H_32_O_16_	MeOH	IR, MS, NMR	[34]
156	quercetin 3-*O*-methyl ether	C_16_H_12_O_7_	MeOH	IR, MS, NMR	[34]
157	kaempferol 3,7,4′-trimethylether	C_18_H_16_O_6_	MeOH	IR, MS, NMR	[34]
158	(–)-loliolide	C_11_H_16_O_3_	MeOH	MS, NMR	[33]
159	*β*-sitosterol	C_29_H_50_O	MeOH	IR, MS, NMR	[34]
160	stigmasterol	C_29_H_48_O	MeOH	IR, MS, NMR	[34]
161	stigmasterol 3-*O*-*β*-D-glucopyranoside	C_31_H_50_O_3_	MeOH	IR, MS, NMR	[34]
162	*β*-sitosterol 3-*O*-*β*-D-glucopyranoside	C_35_H_60_O_6_	MeOH	IR, MS, NMR	[34]

ESIMS: Electrospray Ionization Mass Spectrometry. NMR: Nuclear Magnetic Resonance. IR: Infrared Spectroscopy. HRCIMS: High Resolution Chemical Ionization Mass Spectrometry. MS: Mass Spectrometry. UV: Ultraviolet. HRESIMS: High Resolution Electrospray Ionization Mass Spectrometry. HPLC: High Performance Liquid Chromatography. LC–MS: Liquid Chromatography–Mass Spectrometry. GC-MS/MS: Gas Chromatography–Tandem Mass Spectrometry.

**Table 2:** Pharmacological activity of compounds from *Eupatorium fortunei* Turcz.

No.	Sources	Pharmacological Activities	Types	Models or Test System	Indicated Concentrations	Action or Mechanism	Ref.
1	water extract	Anti-cancer	In vitro	B16F10, HT1080, PC-3, HUVE cells	25, 50, 100 μg/mL	MMP-9 ↓, NF-κB ↓, p38 ↓, JNK ↓, and reduce VEGF via HIF-1*α*/Akt/mTOR pathway	[24]
2	eupatodibenzofuran A	Anti-cancer	In vitro	A549 and MCF-7 cells	3.125, 6.25, 12.5, 25, 50, 100 μM	A549 (IC_50_: 5.95 ± 0.89 µM), MCF-7 (IC_50_: 5.55 ± 0.23 µM), colony-formation: 1.25–10 µM, apoptosis assay: 5, 10 µM, and Western blot: 1.25–10 µM	[30]
3	eupatodibenzofuran B	Anti-cancer	In vitro	A549 and MCF-7 cells	3.125, 6.25, 12.5, 25, 50, 100 μM	A549 (IC_50_: 93.37 ± 1.14 µM), MCF-7 (IC_50_: 85.91 ± 3.94 µM)	[30]
4	6-acetyl-8-methoxy-2,2-dimethylchroman-4-one	Anti-cancer	In vitro	A549 and MCF-7 cells	3.125, 6.25, 12.5, 25, 50, 100 μM	A549 (IC_50_: >100 µM), MCF-7 (IC_50_: >100 µM)	[30]
5	eupatofortunone	Anti-cancer	In vitro	A549 and MCF-7 cells	3.125, 6.25, 12.5, 25, 50, 100 μM	A549 (IC_50_: 86.63 ± 10.89 µM), MCF-7 (IC_50_: 82.15 ± 8.26 µM)	[30]
6	eupatodithiecine	Anti-cancer	In vitro	A549 and MCF-7 cells	3.125, 6.25, 12.5, 25, 50, 100 μM	A549 (IC_50_: 39.44 ± 2.81 µM), MCF-7 (IC_50_: 31.20 ± 4.23 µM)	[30]
7	thymyl angelate	Anti-cancer	In vitro	A549 and MCF-7 cells	3.125, 6.25, 12.5, 25, 50, 100 μM	A549 (IC_50_: >100 µM), MCF-7 (IC_50_: >100 µM)	[30]
8	8,9-dehydrothymol 3-*O*-tiglate	Anti-cancer	In vitro	A549 and MCF-7 cells	3.125, 6.25, 12.5, 25, 50, 100 μM	A549 (IC_50_: >100 µM), MCF-7 (IC_50_: >100 µM)	[30]
9	9-angeloyloxythymol	Anti-cancer	In vitro	A549 and MCF-7 cells	3.125, 6.25, 12.5, 25, 50, 100 μM	A549 (IC_50_: 60.08 ± 3.39 µM), MCF-7 (IC_50_: 52.11 ± 2.16 µM)	[30]
10	9-*O*-angeloyl-8,10-dehydrothymol	Anti-cancer	In vitro	A549 and MCF-7 cells	3.125, 6.25, 12.5, 25, 50, 100 μM	A549 (IC_50_: 55.36 ± 0.80 µM), MCF-7 (IC_50_: 51.70 ± 0.48 µM)	[30]
11	2-hydroxy-4-methylacetophenone	Anti-cancer	In vitro	A549 and MCF-7 cells	3.125, 6.25, 12.5, 25, 50, 100 μM	A549 (IC_50_: >100 µM), MCF-7 (IC_50_: >100 µM)	[30]
12	trans-*O*-Coumaric acid	Anti-cancer	In vitro	A549 and MCF-7 cells	3.125, 6.25, 12.5, 25, 50, 100 μM	A549 (IC_50_: >100 µM), MCF-7 (IC_50_: >100 µM)	[30]
13	6-hydroxy-7-methoxy-2-isopropenyl-5-acetylcumaran	Anti-cancer	In vitro	A549 and MCF-7 cells	3.125, 6.25, 12.5, 25, 50, 100 μM	A549 (IC_50_: 73.97 ± 2.88 µM), MCF-7 (IC_50_: 72.67 ± 3.51 µM)	[30]
14	2,4-di-tert-butylphenol	Anti-cancer	In vitro	A549 and MCF-7 cells	3.125, 6.25, 12.5, 25, 50, 100 μM	A549 (IC_50_: >100 µM), MCF-7 (IC_50_: >100 µM)	[30]
15	1-(2-hydroxy-5-methoxy-4-methylphenyl) ethanone	Anti-cancer	In vitro	A549 and MCF-7 cells	3.125, 6.25, 12.5, 25, 50, 100 μM	A549 (IC_50_: >100 µM), MCF-7 (IC_50_: >100 µM)	[30]
16	coumarin	Anti-cancer	In vitro	A549 and MCF-7 cells	3.125, 6.25, 12.5, 25, 50, 100 μM	A549 (IC_50_: >100 µM), MCF-7 (IC_50_: >100 µM)	[30]
17	taraxasterol	Anti-cancer	In vitro	A549 and MCF-7 cells	3.125, 6.25, 12.5, 25, 50, 100 μM	A549 (IC_50_: 94.79 ± 10.23 µM), MCF-7 (IC_50_: 93.59 ± 6.11 µM)	[30]
18	9-angeloyloxy-8,9-dehydrothymol	Anti-cancer	In vitro	MCF-7, HeLa, A549, HepG2 cells	N.D.	MCF-7 (IC_50_: 8.42 ± 1.42 µM), HeLa (IC_50_: 6.65 ± 2.34 µM), A549 (IC_50_: 8.78 ± 3.20 µM), HepG2 (IC_50_: 10.40 ± 2.83 µM)	[32]
19	9-(3-methyl-2-butenoyloxy)-8,10-dehydrothymol	Anti-cancer	In vitro	MCF-7, HeLa, A549, HepG2 cells	N.D.	MCF-7 (IC_50_: 6.24 ± 1.38 µM), HeLa (IC_50_: 7.97 ± 3.16 µM), A549 (IC_50_: 9.84 ± 1.96 µM), HepG2 (IC_50_: 11.96 ± 2.80 µM)	[32]
20	7-isobutyryloxythymol	Anti-cancer	In vitro	MCF-7, HeLa, A549, HepG2 cells	N.D.	MCF-7 (IC_50_: >50 µM), HeLa (IC_50_: >50 µM), A549 (IC_50_: >50 µM), HepG2 (IC_50_: >50 µM)	[32]
21	7-isobutyryloxy-8,9-dehydrothymol	Anti-cancer	In vitro	MCF-7, HeLa, A549, HepG2 cells	N.D.	MCF-7 (IC_50_: >50 µM), HeLa (IC_50_: >50 µM), A549 (IC_50_: >50 µM), HepG2 (IC_50_: >50 µM)	[32]
22	2-acetyl-7-tigloyloxy-isothymol	Anti-cancer	In vitro	MCF-7, HeLa, A549, HepG2 cells	N.D.	MCF-7 (IC_50_: >50 µM), HeLa (IC_50_: >50 µM), A549 (IC_50_: >50 µM), HepG2 (IC_50_: >50 µM)	[32]
23	9-*O*-angeloyl-8,10-dehydrothymol	Anti-cancer	In vitro	MCF-7, HeLa, A549, HepG2 cells	N.D.	MCF-7 (IC_50_: 8.17 ± 2.01 µM), HeLa (IC_50_: 6.87 ± 2.97 µM), A549 (IC_50_: 9.72 ± 2.88 µM), HepG2 (IC_50_: 9.23 ± 1.31 µM)	[32]
24	14-hydroxy-8*β*-[4′-hydroxytigloyloxy]-costunolide	Anti-cancer	In vitro	PC3, SKOV3, A549, MCF-7, HEp-2 cells	1–100 μg/mL	PC3 (IC_50_: 15.4 ± 1.3 μM), SKOV3 (IC_50_: ≥100 μM), A549 (IC_50_: ≥100 μM), MCF-7 (IC_50_: 13.5 ± 3.5 μM), HEp-2 (IC_50_: 56.3 ± 3.2μM)	[35]
25	14-acetoxy-8*β*-[4′-hydroxytigloyloxy]-costunolide	Anti-cancer	In vitro	PC3, SKOV3, A549, MCF-7, HEp-2 cells	1–100 μg/mL	PC3 (IC_50_: 4.2 ± 1.3 μM), SKOV3 (IC_50_: 32.5 ± 7.4 μM), A549 (IC_50_: 28.3 ± 0.9 μM), MCF-7 (IC_50_: 6.1 ± 1.8 μM), HEp-2 (IC_50_: 20.9 ± 6.2 μM)	[35]
26	14-acetoxy-8*β*-hydroxy-costunolide	Anti-cancer	In vitro	PC3, SKOV3, A549, MCF-7, HEp-2 cells	1–100 μg/mL	PC3 (IC_50_: 15.2 ± 1.0 μM), SKOV3 (IC_50_: 46.0 ± 6.8 μM), A549 (IC_50_: 52.3 ± 8.0 μM), MCF-7 (IC_50_: 30.0 ± 7.4 μM), HEp-2 (IC_50_: 54.9 ± 3.0 μM)	[35]
27	8*β*-[4′,5′-dihydroxytigloyloxy]-costunolide	Anti-cancer	In vitro	PC3, SKOV3, A549, MCF-7, HEp-2 cells	1–100 μg/mL	PC3 (IC_50_: 3.9 ± 1.2 μM), SKOV3 (IC_50_: 3.8 ± 6.0 μM), A549 (IC_50_: 37.9 ± 7.5 μM), MCF-7 (IC_50_: 12.8 ± 3.3 μM), and HEp-2 (IC_50_: 26.4 ± 6.5μM)	[35]
28	8*β*-[4′-hydroxytigloyloxy]-14-oxo-costunolide	Anti-cancer	In vitro	PC3, SKOV3, A549, MCF-7, HEp-2 cells	1–100 μg/mL	PC3 (IC_50_: 12.6 ± 4.0 μM), SKOV3 (IC_50_: 31.8 ± 4.6 μM), A549 (IC_50_: 25.9 ± 1.0 μM), MCF-7 (IC_50_: 16.3 ± 3.4 μM), HEp-2 (IC_50_: 25.0 ± 7.1 μM)	[35]
29	3*β*-acetoxy-8*β*-[4′,5′-dihydroxytigloyloxy]-costunolide	Anti-cancer	In vitro	PC3, SKOV3, A549, MCF-7, HEp-2 cells	1–100 μg/mL	PC3 (IC_50_: 17.4 ± 2.3 μM), SKOV3 (IC_50_: 68.6 ± 2.2 μM), A549 (IC_50_: 61.2 ± 3.3 μM), MCF-7 (IC_50_: 28.9 ± 1.2 μM), HEp-2 (IC_50_: 26.8 ± 1.1 μM)	[35]
30	2*β*-hydroxy-8*β*-[5′-hydroxytigloyloxy]-costunolide	Anti-cancer	In vitro	PC3, SKOV3, A549, MCF-7, HEp-2 cells	1–100 μg/mL	PC3 (IC_50_: 8.1 ± 0.3 μM), SKOV3 (IC_50_: 9.4 ± 2.9 μM), A549 (IC_50_: 29.3 ± 7.9 μM), MCF-7 (IC_50_: 5.8 ± 0.1 μM), HEp-2 (IC_50_: 27.6 ± 3.4 μM)	[35]
31	1*β*-hydroxy-8*β*-[4′-hydroxytigloyloxy]-*β*-cyclocostunolide	Anti-cancer	In vitro	PC3, SKOV3, A549, MCF-7, HEp-2 cells	1–100 μg/mL	PC3 (IC_50_: 3.9 ± 0.6 μM), SKOV3 (IC_50_: 36.9 ± 2.5 μM), A549 (IC_50_: 44.8 ± 6.1 μM), MCF-7 (IC_50_: 8.3 ± 0.9 μM), HEp-2 (IC_50_: 22.7 ± 0.3 μM)	[35]
32	1*β*-hydroxy-8*β*-[4′-hydroxytigloyloxy]-*α*-cyclocostunolide	Anti-cancer	In vitro	PC3, SKOV3, A549, MCF-7, HEp-2 cells	1–100 μg/mL	PC3 (IC_50_: 4.7 ± 0.9 μM), SKOV3 (IC_50_: 50.2 ± 6.5 μM), A549 (IC_50_: 45.6 ± 3.6 μM), MCF-7 (IC_50_: 14.6 ± 5.0 μM), HEp-2 (IC_50_: 25.6 ± 1.2 μM)	[35]
33	8*β*-[4′-hydroxytigloyloxy]-costunolide	Anti-cancer	In vitro	PC3, SKOV3, A549, MCF-7, HEp-2 cells	1–100 μg/mL	PC3 (IC_50_: 4.3 ± 0.8 μM), SKOV3 (IC_50_: 20.2 ± 5.0 μM), A549 (IC_50_: 17.8 ± 3.8 μM), MCF-7 (IC_50_: 6.1 ± 0.3 μM), HEp-2 (IC_50_: 12.6 ± 2.7 μM)	[35]
34	water extract	Anti-viral	In vitro	RAW 264.7 cells and viral infection (e.g., influenza virus)	100 μg/mL	Induces IFN-*β*, IL-6, and TNF-α. IRF3↑, STAT1↑, TBK1↑, and reduces viral replication	[52]
35	quercetin	Anti-viral	In vitro	RAW 264.7 cells and viral infection (e.g., influenza virus)	0.2 μg/mL	Inhibition of viral gene expression, replication, mRNA synthesis, and induction of immune responses	[52]
36	psoralen	Anti-viral	In vitro	RAW 264.7 cells and viral infection (e.g., influenza virus)	0.2 μg/mL	Inhibition of viral gene expression and replication	[52]
37	thymol	Anti-microbial	In vitro	Fungi (e.g., Fusarium oxysporum)	10 mg/mL	Disrupts cell wall, membrane integrity, and MIC: 0.12–0.31 mg/mL	[12]
38	*β*-caryophyllene	Anti-microbial	In vitro	Fungi (e.g., Fusarium oxysporum)	10 mg/mL	Disrupts cell membrane permeability, and MIC: 0.11–1.07 mg/mL	[12]
39	(+)-eupafortunin F	Anti-inflammatory	In vitro	LPS-stimulated RAW264.7 cells	1.56–100 μM	NO ↓ and NO (IC_50_: 25.04 ± 1.37 µM)	[3]
40	(−)-eupafortunin F	Anti-inflammatory	In vitro	LPS-stimulated RAW264.7 cells	1.56–100 μM	NO ↓ and NO (IC_50_: 24.12 ± 1.25 µM)	[3]
41	eupafortunin M	Anti-inflammatory	In vitro	LPS-stimulated RAW264.7 cells	1.56–100 μM	NO ↓ and NO (IC_50_: 27.19 ± 0.93 µM)	[3]
42	eupafortunin N	Anti-inflammatory	In vitro	LPS-stimulated RAW264.7 cells	1.56–100 μM	NO ↓ and NO (IC_50_: 20.98 ± 0.38 µM)	[3]
43	(+)-9-angeloyloxy-8,10-dihydroxythymol	Anti-inflammatory	In vitro	LPS-stimulated RAW264.7 cells	1.56–100 μM	NO ↓ and NO (IC_50_: 41.17 ± 4.59 µM)	[3]
44	(−)-9-angeloyloxy-8,10-dihydroxythymol	Anti-inflammatory	In vitro	LPS-stimulated RAW264.7 cells	1.56–100 μM	NO ↓ and NO (IC_50_: 37.64 ± 0.60 µM)	[3]
45	7-hydroxythymol	Anti-inflammatory	In vitro	LPS-stimulated RAW264.7 cells	1.56–100 μM	NO ↓ and NO (IC_50_: 44.07 ± 1.87 µM)	[3]
46	2-acetyl-3-methoxy-5-(prop-1-ynyl)-thiophene	Anti-inflammatory	In vitro	RAW264.7 and Beas-2B cells	1.56–100 μM	Nrf2 ↑, HO-1 ↑, GCLM↑, NQO1 ↑, ROS ↓, NO ↓, NO (IC_50_: 18.24 ± 1.94 µM), Nrf2 (IC_50_: 40–80 µM)	[3]
47	1-monooleoylglycerol	Anti-inflammatory	In vitro	LPS-stimulated RAW264.7 cells	>0.5 mg	NO ↓ and NO (IC_50_: 20.99 ± 0.19 µM)	[4]
48	brachangelonina	Anti-inflammatory	In vitro	LPS-stimulated RAW264.7 cells	>0.5 mg	NO ↓, ROS ↓, iNOS ↓, COX-2 ↓, NF-κB ↓, nuclear translocation, and NO (IC_50_: 5.67 ± 0.37 µM)	[4]
49	luteolin	Anti-inflammatory	In vitro	Network pharmacology	N.D.	TLR4/JNK ↓, *α*-glucosidase ↓, IL-6 ↓, TNF-*α* ↓	[53]
50	luteolin	Anti-diabetic	In vitro	Network pharmacology	N.D.	TLR4/JNK ↓ *α*-glucosidase ↓, IL-6 ↓, TNF-*α* ↓	[53]
51	9-acetoxyl-3-isobutyroylthymol	Anti-diabetic	In vitro	*α*-glucosidase	1–256 μg/mL	Inhibition (5–14%)	[31]
52	9-acetoxythymol 3-*O*-tiglate	Anti-diabetic	In vitro	*α*-glucosidase	1–256 μg/mL	Inhibition (5–14%)	[31]
53	9-acetoxy-8,10-dehydrothymol 3-*O*-tiglate	Anti-diabetic	In vitro	*α*-glucosidase	1–256 μg/mL	Inhibition (5–14%)	[31]
54	eupafortuni C	Anti-oxidant	In vitro	2,2′-azinobis (3-ethylbenzothiazoline-6-sulphonic acid	1.56–100 μM	Radical scavenging and anti-radical (IC_50_: 24.27 ± 8.12 µM)	[3]
55	(+)-9-hydroxythymol	Anti-oxidant	In vitro	2,2′-azinobis (3-ethylbenzothiazoline-6-sulphonic acid	1.56–100 μM	NO ↓ and anti-radical (IC_50_: 22.05 ± 3.22 µM)	[3]
56	(−)-9-hydroxythymol	Anti-oxidant	In vitro	2,2′-azinobis (3-ethylbenzothiazoline-6-sulphonic acid	1.56–100 μM	Radical scavenging and anti-radical (IC_50_: 10.42 ± 0.83 µM)	[3]
57	(−)-methyl 2-(2-hydroxy-4-methylphenyl) propanoate	Anti-oxidant	In vitro	2,2′-azinobis (3-ethylbenzothiazoline-6-sulphonic acid	1.56–100 μM	Radical scavenging and anti-radical (IC_50_: 14.14 ± 3.80) µM	[3]
58	(4*β*,6*β*)-6-hydroxypiperitone	Anti-oxidant	In vitro	2,2′-azinobis (3-ethylbenzothiazoline-6-sulphonic acid	1.56–100 μM	Radical scavenging and anti-radical (IC_50_: 8.89 ± 0.61 µM)	[3]
59	2-acetyl-3-methoxy-5-(prop-1-ynyl)-thiophene	Anti-oxidant	In vitro	2,2′-azinobis (3-ethylbenzothiazoline-6-sulphonic acid, RAW264.7 and Beas-2B cells	1.56–100 μM	Nrf2 ↑, HO-1 ↑, GCLM↑, NQO1 ↑, ROS ↓, NO ↓, NO (IC_50_: 18.24 ± 1.94 µM) and Nrf2 (IC_50_: 40–80 µM)	[3]
60	7,8,9-trihydroxythymol	Anti-cyanobacterial	In vitro	Microcystis aeruginosa	50 μg/mL	N.D.	[28]
61	8,10-didehydro-7,9-dihydroxythymol	Anti-cyanobacterial	In vitro	Microcystis aeruginosa	50 μg/mL	Inhibition (39.1%)	[28]
62	8,9,10-trihydroxythymol	Anti-cyanobacterial	In vitro	Microcystis aeruginosa	50 μg/mL	Inhibition (45.6%), comparable to CuSO_4_ (47.5% at 5 μg/mL) and anti-cyanobacterial (IC_50_: 62.4 ± 8.3 μg/mL)	[28]
63	10-acetoxy-8,9-dihydroxythymol	Anti-cyanobacterial	In vitro	Microcystis aeruginosa	50 μg/mL	Inhibition (43.1%) and anti-cyanobacterial (IC_50_: 73.7 ± 12.6 μg/mL)	[28]
64	*O*-coumaric acid	Anti-cyanobacterial	In vitro	Microcystis aeruginosa	50 μg/mL	N.D.	[28]
65	4-(2-hydroxyethyl) benzaldehyde	Anti-cyanobacterial	In vitro	Microcystis aeruginosa	50 μg/mL	Inhibition (43.0%)	[28]
66	9-acetoxyl-3-isobutyroylthymol	Anti-acetylcholinesterase	In vitro	5-thio-2-nitrobenzoate	1–256 μg/mL	Inhibition (17–25%)	[31]
67	9-acetoxythymol 3-*O*-tiglate	Anti-acetylcholinesterase	In vitro	5-thio-2-nitrobenzoate	1–256 μg/mL	Inhibition (17–25%)	[31]
68	9-acetoxy-8,10-dehydrothymol 3-*O*-tiglate	Anti-acetylcholinesterase	In vitro	5-thio-2-nitrobenzoate	1–256 μg/mL	Inhibition (17–25%)	[31]
69	methanol extract	Anthelmintic activity	In vivo	Goldfish with Dactylogyrus intermedius	500 mg/L	Inhibition (100%)	[54]

*↑*: Promote, *↓*: Inhibition.

## Data Availability

No new data were created or analyzed in this study. Data sharing is not applicable to this article.
